# Amalgam *Versus* Gold

**Published:** 1894-05

**Authors:** George A. Mills

**Affiliations:** New York


### AMALGAM *VERSUS* GOLD.

BY DR. GEORGE A. MILLS, NEW YORK.

We judge that, after forty-two years of more than ordinary interest in dental practice, we are prepared to express our thoughts upon this subject. Those who know us will give credit for following what we believe to be our convictions. No one has more earnestly or more faithfully advocated the importance of our calling, and it is because of the increased value of its importance that we propose to put ourselves on record regarding a duty towards those that as sincerely seek at our hands the services they need, as we all sincerely desire to give these services. If dental practice means more one thing than another, the emphasis should be put upon the thought of saving teeth in as large a percentage as possible, and this with the smallest expenditure of time, money, and nervous force. We are prepared to say that the last thirty years has a large account against us, and we think that the next thirty should be as devotedly used for the cancelling of this account. It is only necessary to hint at the experience of all earnest operators who have followed close upon us during this term of years. To say it has been a burden of great expense to us all, both patient and operator, is only saying what all know to be true.

Truly it can be characterized as the Golden Age. It has been said by some to have been the "Gold craze." No, not a craze in any sense, but as sincere a desire as ever possessed any class of men to perform their whole duty. The use of gold for filling teeth has been carried to its fullest extreme. Atkinson, Varney, Webb, and Brown will stand among the notables in this line of service. They have shown us the perfection with which this metal can be handled, even to the ideal, both in its thoroughness of compact and its correspondence to Nature's forms and contact, giving back to the possessor of the sadly deformed masticating apparatus as comfortable and useful a reconstruction as it is possible for a man to devise with this material upon Nature's foundations.

Have they done all this amiss? Certainly not. They have acted up to their best light considering the human entanglements that have involved their career. We allude to this that it may not be said, with any effective force, "I told you so." We are not in sympathy at all with the weak and unwise criticism, even if it can be called criticism, that has come from not a few worthy men regarding the handiwork of these extreme and skilful operators. Every one of them has emphasized excellence, which has a decided bearing upon all the service that we are called upon to render. Everything that is worth doing commands our best attention, and let it be understood that we have in mind only those who have a conscientious desire for the good of those who place themselves under their care. Let us ask a question that will appeal to all upright practitioners. Have you not frequently been brought face to face—in view of a lamentable failure—with this thought? Would I not, in view of what I now see, and in case I could retrace my steps, change my dealings with a hope of far different results, and give to my patient a service much larger in proportion? How many of us are always manly enough to meet such a demand squarely and fairly, and say, I have learned a lesson that shall stand for all time? These experiences do, more or less, influence our course, but none of us who are really skilful come down easily. Humility is not a crowning virtue. It is only when a bold stroke comes like that applied by our genial Foster Flagg that we become reflective, and not then while under the first effect of the lash.

Since that time we have had opportunity to reflect; and we have reflected, and to all men of judgment this has brought about a decidedly eclectic procedure, and a larger range of conservative practice; while, on the other hand, it has afforded an occasion for others to cater unwisely to the weaknesses of a clientele of the so-called *élite*, which has not proved economical in time, money, or saving of teeth. These things are so well understood by observing men that we need only refer to the fact. It is not necessary to recapitulate what is well known by all readers of our journals,—that the "causes of failure in filling teeth" has become a hackneyed topic, without really touching the principles that lead to these prolific failures that are occurring on all hands to a greater or less degree. True, we are under human limitations, but to say that we have reached the largest possibilities in saving teeth, even with the knowledge in our hands, is not squaring ourselves to the facts.

At this stage of our article we are prepared to say intelligently, and without any fear of the contrary being proved, that we have in our hands both knowledge and an ally that will enable us to so change our practice that we can increase the salvatory service—which is so much needed—in a very large percentage above what we have previously been able to do. We do not advocate the practice that we are aiming at in this article because of the inability of the many to save teeth during long terms of years, but it is because we are facing well-considered convictions that we can do all this and much more with far less expense to all parties concerned.

One of the most painstaking operators in Brooklyn, and none more skilful, said lately, showing some very elaborate fillings in the molars and bicuspids made under great difficulties, but successful now for a period of twenty-three years, "To-day I would not repeat such an effort. I can do as well with a great reduction of cost." Said he, "I put my very life into that work, and no man can ever hope to be paid a fair compensation for such service."

No one, for a moment, would hesitate to laud the discovery of a gold plastic for filling, but what will many say when the fact is stated that we have a plastic metal filling that will meet the larger demands with a greater percentage of success directly at hand? Does some one say now, with bated breath, that we are to be faced with the advocacy of the so-called "Amalgam *versus* Gold"? Yes, for we have never made an effort with firmer purpose than this or more faith that it will result in fruitage. We know intuitively that many are occupying their thought in the same direction. This reform of our practice is a foregone conclusion, and the way to bring it about is to openly advocate it.

Let it be understood that we frankly and understandingly declare it to be our discriminating belief that amalgam, as it is offered to us to-day, has in it the largest possibilities of any of the other materials. We hardly need to call attention to the former objections so far as discoloring, for this material is now free from that defect. The shrinking and bulging is not worthy of intelligent attention, and so far as the objection made to its deleterious effect upon the system, it stands "not proven."

Now that we have unmasked our purpose, we will endeavor to lead the attention to the fact that must enlist the emphasis upon two essential additional points of practice, in order that our statements may be proved to the fullest extent. Nothing but real ability will bring the largest amount of success. The first of these essential points is the securing of the best possible sanitary conditions.

Antiseptic lotions and tooth-brushes, and here and there atomizing of a numberless variety of medicaments, will not meet the demands of a majority of cases. Nothing but a radical intelligent dealing will be efficacious. Having secured this, we have reduced the exciting cause of caries to a minimum.

Now we are prepared for the next essential step,—*i.e.*, the prepation of the tooth for receiving the filling-material. I only advocate what others have done, bestow the same care and thoroughness in the one case as in the other. When practitioners have more largely devoted their faithful attention to these all-essential points, we will then have far less cause to discuss why our fillings fail. Carry out these teachings, and they will do more to aid the overworked practitioner and bring from our patients such plaudits of praise as we have never conceived. If you believe from your heart, through your head, that the declaration of this is true, there will be but little difficulty in carrying it into practice. If the truth of this article is recognized, many must inevitably adopt it.

We will close by prophesying that gold will have to give way to amalgam and acknowledge its superiority. Nothing stands in the way of its rightful position but prejudiced thought formed by ignorance and past associations. We have done much in the filling of teeth in past days along all the most advanced lines.

Our practice has so changed during the last fifteen years that we have been largely spared the laborious work of dealing with extensive and difficult operations in filling. Our surgical work has largely taken the place of this line of service. We are content to believe that we are much relieved from the arduous labor of this branch of practice. We have done many things under the conviction that we were advocating for the good of our fellows, and none more so than this. That we may have the best material possible, we hail it a favorable omen that Dr. Morgan Howe has called the attention of the Odonotological Society of New York to the great need of taking steps for securing the best formulæ for amalgam, and this by the employment of expert scientific ability, outside the profession if required. The day will come when we will acknowledge our indebtedness to Dr. Clowes, of New York, for his abiding faith in the ultimate recognition of the value of amalgam. He has stood steadfast even to date, and there has been no more honest or able advocate, and all his practice has had the stamp of integrity upon it. It is very largely because amalgam has been made a substitute that it has had very indifferent consideration so far as associating skill with its use.

We are not declaring that gold is not to have any place as a filling-material, but we do declare that it cannot be made to serve the purpose of saving the greater number of teeth that most need saving. We have emphasized the importance of securing for the teeth the best possible sanitary conditions, and the most skilful preparation for filling, and we add to this the same painstaking in finishing as we give to our most elaborate gold fillings. Then impress our patients with the responsibility that rests upon them for their proper care. We have for many years advocated that indifference to sanitary conditions stood against the mass of practitioners as the flagrant cause of failure of fillings, and we can truthfully say that to this must be added indifferent attention to the care of the teeth attacked by caries. To be sure, we cannot guarantee against more or less deformation occurring, though we do our best. There is a larger helpfulness of an advance in a salvatory service if these views here given shall be put into practical use, and we are also sure that our patients will be ready to accept the purpose of our earnest and intelligent service.

